# 68 Using the Cornell Assessment of Pediatric Delirium Score to Identify Delirium in Pediatric Burn Patients

**DOI:** 10.1093/jbcr/irad045.042

**Published:** 2023-05-15

**Authors:** Katherine Bergus, Tran Bourgeois, Kelli Patterson, Dana Schwartz, Rajan Thakkar, Renata Fabia

**Affiliations:** Nationwide Children's Hospital & The Ohio State University, Columbus, Ohio; The Abigail Wexner Research Institute at Nationwide Children's Hospital, Columbus, Ohio; Nationwide Children’s Hospital; Ohio State University, Columbus, Ohio; Nationwide Children's Hospital, Columbus, Ohio; Nationwide Children's Hospital, Columbus, Ohio; Nationwide Children's Hospital, Columbus, Ohio; Nationwide Children's Hospital & The Ohio State University, Columbus, Ohio; The Abigail Wexner Research Institute at Nationwide Children's Hospital, Columbus, Ohio; Nationwide Children’s Hospital; Ohio State University, Columbus, Ohio; Nationwide Children's Hospital, Columbus, Ohio; Nationwide Children's Hospital, Columbus, Ohio; Nationwide Children's Hospital, Columbus, Ohio; Nationwide Children's Hospital & The Ohio State University, Columbus, Ohio; The Abigail Wexner Research Institute at Nationwide Children's Hospital, Columbus, Ohio; Nationwide Children’s Hospital; Ohio State University, Columbus, Ohio; Nationwide Children's Hospital, Columbus, Ohio; Nationwide Children's Hospital, Columbus, Ohio; Nationwide Children's Hospital, Columbus, Ohio; Nationwide Children's Hospital & The Ohio State University, Columbus, Ohio; The Abigail Wexner Research Institute at Nationwide Children's Hospital, Columbus, Ohio; Nationwide Children’s Hospital; Ohio State University, Columbus, Ohio; Nationwide Children's Hospital, Columbus, Ohio; Nationwide Children's Hospital, Columbus, Ohio; Nationwide Children's Hospital, Columbus, Ohio; Nationwide Children's Hospital & The Ohio State University, Columbus, Ohio; The Abigail Wexner Research Institute at Nationwide Children's Hospital, Columbus, Ohio; Nationwide Children’s Hospital; Ohio State University, Columbus, Ohio; Nationwide Children's Hospital, Columbus, Ohio; Nationwide Children's Hospital, Columbus, Ohio; Nationwide Children's Hospital, Columbus, Ohio; Nationwide Children's Hospital & The Ohio State University, Columbus, Ohio; The Abigail Wexner Research Institute at Nationwide Children's Hospital, Columbus, Ohio; Nationwide Children’s Hospital; Ohio State University, Columbus, Ohio; Nationwide Children's Hospital, Columbus, Ohio; Nationwide Children's Hospital, Columbus, Ohio; Nationwide Children's Hospital, Columbus, Ohio

## Abstract

**Introduction:**

Delirium rates in pediatric critical care range from 18-40% and its development is associated with baseline cognitive dysfunction, primary diagnosis, and mechanical ventilation. The Cornell Assessment of Pediatric Delirium (CAPD) is a tool used to detect delirium in children of all ages, but has not been validated in burn patients. Our study aimed to use CAPD score to determine the frequency of delirium in pediatric burn patients and assess the association of delirium with burn demographics.

**Methods:**

We conducted a retrospective review of patients aged 2.5 weeks to 18 years who were admitted to our ABA-verified pediatric burn center from 2018-2021 and who underwent delirium screening with the CAPD tool (CAPD ≥9 indicates a diagnosis of delirium). Injury mechanism, patient demographics, hospitalization details, and CAPD score were collected and χ^2^, Fisher’s exact test, and univariate analyses performed.

**Results:**

389 patients with documented CAPD scores were included in our cohort, with median age of 2 years at the time of burn injury. Delirium was identified in 42 (10.8%) patients. Delirious patients were generally older compared to non-delirious patients (4 years (IQR 2, 11) vs 2 years (IQR 1, 6), *P *< 0.0005) and had higher TBSA burned (21.63% (IQR 9, 42) vs 3.5% (IQR 1.75, 6), *P* < 0.0001). Odds of developing delirium with increasing age was 1.08 (95% CI: 1.02-1.15, *P* = 0.0068) and 1.16 (95% CI: 1.11-1.20, *P* = < 0.0001) for each percent increase in TBSA burned. Delirium diagnosis did not vary significantly with gender or race/ethnicity. Though it did not reach statistical significance, percent probability of developing delirium was highest among fire burns (26.53% (95% CI: 17.79-35.27)), and lowest among chemical burns (1.49% (95%CI: 0-4.40), *P* > 0.05). Patients who underwent 6 or more Anesthesia Events (AE) were 74.43 times more likely to develop delirium than patients who underwent 0 AEs (*P *< 0.001). Compared to non-delirious patients, patients with delirium were 81.02 times more likely to have an ICU admission (95% CI: 38.79-169.22, *P* < 0.0001) and 11.37 times more likely to have a longer hospital admission (95% CI: 7.74-16.72, *P* < 0.0001).

**Conclusions:**

Screening with the CAPD tool identified delirium in pediatric burn patients with known risk factors for delirium including higher TBSA burned, greater number of AEs, and longer ICU course or hospital admission. Delirium increased with age in our study group. There are few validated tools for measuring delirium in pediatric burn patients, and further studies are needed determine whether CAPD accurately captures delirium in younger burn patients.

**Applicability of Research to Practice:**

Knowing associations between clinical burn characteristics and delirium can help predict which patients are at highest risk. Early identification of these patients can enable extra precautions to prevent delirium and to treat it early if it develops.

